# Risk factors for incident type 2 diabetes among adults with impaired fasting glucose: a comparison of three regression models accounting for competing mortality in Liuzhou, Guangxi, China

**DOI:** 10.1016/j.pmedr.2026.103616

**Published:** 2026-09-01

**Authors:** Zhenzhen Lu, Chaoyun Li, Qi Li, Jun Lv, Canqing Yu, Dianjianyi Sun, Wei Mao

**Affiliations:** a Guangxi Zhuang Autonomous Region Center for Disease Control and Prevention, Nanning 530028, PR China; b Guangxi Zhuang Autonomous Region Health Statistics Information Center, Nanning 530028, PR China; c School of Information and Management, Guangxi Medical University, Nanning 530021, PR China; d Department of Epidemiology & Biostatistics, School of Public Health, Peking University, Xueyuan Road, Haidian District, Beijing 100191, PR China; e Peking University Center for Public Health and Epidemic Preparedness and Response, Beijing 100191, PR China; f Key Laboratory of Epidemiology of Major Diseases (Peking University), Ministry of Education, Beijing 100191, PR China

**Keywords:** Type 2 diabetes, Impaired fasting glucose, Competing risk analysis, Cox regression, Logistic regression, Anthropometric biomarkers

## Abstract

**Objectives:**

Impaired fasting glucose (IFG) is a prediabetic precursor of type 2 diabetes (T2DM). Few long-term cohorts with substantial competing mortality compare risk estimates from logistic, Cox and Fine-Gray regression. This study aimed to examine cross-model discrepancies and demonstrate model-dependent bias in IFG risk inference.

**Methods:**

We followed 913 Chinese IFG adults recruited in Liuzhou, Guangxi (2004–2008) for up to 20 years until 31 December 2024 (median 17.83 years). Three regression models were used to identify fully adjusted T2DM predictors.

**Results:**

Fasting glucose, body fat percentage and older age were consistent positive predictors; female sex was persistently protective. Body mass index (BMI) ≥28 kg/m^2^ and waist-to-hip ratio showed divergent results across models. Hypertension lacked independent effects in all models, and enrollment year was significant only in Cox regression.

**Conclusions:**

Statistical model choice alters risk factor conclusions in IFG cohorts with high competing mortality. Joint interpretation of complementary regression methods is recommended.

## Highlights

• Risk factor inferences vary with statistical model selection for cohorts with substantial competing-risk mortality.
• Competing-death risk may distort associations of risk factors for incident type 2 diabetes.
• Traditional Cox models may overestimate hazard effects in populations with high mortality risk.

## 1 Introduction

Type 2 diabetes mellitus (T2DM) imposes a substantial global public health burden with rising population prevalence worldwide. Fasting plasma glucose is a low-cost, widely accessible clinical indicator for screening prediabetes and T2DM worldwide (Kohansal et al., 2022). Impaired fasting glucose (IFG) defines a hyperglycemic state above normal thresholds but below the diagnostic cutoff for diabetes (Harland et al., 2025). Adults with IFG carry markedly elevated long-term risk of progressing to T2DM, and identifying predictive biomarkers is critical for targeted early intervention (Dagogo-Jack, 2025; Tabák et al., 2012).

Most prior epidemiological analyses of prediabetes progression relied solely on either multivariate logistic regression or conventional Cox regression, with all-cause mortality frequently ignored as a competing endpoint. Participants who die from any cause cannot develop new-onset diabetes during remaining follow-up; simple administrative censoring of deceased individuals introduces bias to risk estimates (Putter et al., 2007; Andersen et al., 2012). In this 20-year Chinese prospective cohort study, we applied three complementary regression models to compare fully adjusted risk estimates, quantify cross-model discrepancies, and provide methodological references for future prediabetes cohort analyses (Austin and Fine, 2017; Haller et al., 2013).

## 2 Methods

### 2.1 Study design and population

This is an observational retrospective cohort study without interventional procedures; therefore, clinical trial registration was not applicable. Participants were enrolled from the Liuzhou subcohort of the China Kadoorie Biobank (CKB), and full cohort design details have been published previously (Chen et al., 2011). A total of 50,173 adults aged 35–74 years were recruited between October 26, 2004, and July 30, 2008. Individuals with pregnancy, active pulmonary tuberculosis or physical disabilities at baseline were excluded. The follow-up censoring date was set as December 31, 2024, with a maximum follow-up duration of 20 years (median: 17.83 years).

### 2.2 Measures

Standardized physical examinations and venous blood collection were performed by professionally trained physicians at baseline. All participants received random blood glucose testing at baseline. Participants with random blood glucose levels ranging from 7.8 to 11.1 mmol/L and without self-reported diabetes underwent an additional fasting blood glucose assessment. Fasting glucose classification followed American Diabetes Association criteria (*Diabetes Care*, 2024). IFG was defined as fasting plasma glucose 5.6–6.9 mmol/L.

All-cause survival status and date of death were ascertained via the National Mortality Surveillance System and cross-verified with national medical insurance records. Participants with untraceable vital status were excluded, resulting in the exclusion of 62 individuals. Incident type 2 diabetes events were identified through linked multi-source administrative records, including public health service databases, hospital discharge summaries, medical insurance claim records and mortality surveillance data. The date of diabetes diagnosis was defined as the earliest recorded event date across all data sources.

### 2.3 Statistical analysis

Baseline characteristics were described as mean ± standard deviation for normally distributed continuous variables, median (interquartile range) for non-normal continuous variables, and count (percentage) for categorical variables. Group comparisons were performed via Student's *t*-test, Mann-Whitney *U* test or χ^2^ test as appropriate.

Three multivariate regression models with identical covariate adjustment sets were used to identify independent T2DM predictors:

Multivariate binary logistic regression: Static cross-sectional model; binary outcome (1 = incident T2DM, 0 = no T2DM). Results were presented as odds ratio (OR) and corresponding 95% confidence interval (CI).

Cause-specific Cox proportional hazards regression: Time-to-event survival model; all-cause death and loss to follow-up were treated as administrative censoring. Hazard ratio (HR) and 95% CI were calculated after verifying the proportional hazards assumption.

Fine-Gray subdistribution competing risk regression: All-cause mortality was defined as the competing event, and subdistribution hazard ratio (sHR) with 95% CI was estimated to eliminate competing risk bias.

Identical covariates were adjusted across all models, including age category, sex, body mass index (BMI) category, body fat percentage, waist-to-hip ratio, categorized blood pressure, fasting plasma glucose, random plasma glucose, and enrollment year. Two-sided *P* < 0.05 was defined as the statistical significance threshold.

All statistical analyses were performed using R software version 4.5.2.

This sub-cohort analysis received independent ethical clearance from the Ethics Review Committee of Guangxi Zhuang Autonomous Region (approval No. GXCDCIRB 2022-0044-01). This study followed the human-subject protection guidelines of the CKB data curator. All participants provided informed consent, and the analytical dataset used in this study was fully anonymized to protect participant privacy.

## 3 Results

The final analytical cohort included 913 eligible IFG participants. The median baseline age was 57 years (IQR: 52–65), and 61.85% of participants were female. During follow-up, 387 (42.39%) individuals developed incident T2DM, and 245 (26.84%) participants experienced all-cause death.

After identical multivariable adjustment, advanced age (including 45–59 years and ≥60 years), fasting plasma glucose, and body fat percentage displayed consistent significant positive associations with incident T2DM across all three models (all *P* < 0.05). Female sex acted as a stable protective factor in all analytical frameworks (all *P* < 0.05). Categorized systolic and diastolic blood pressure showed no independent correlation with T2DM after full adjustment, despite crude intergroup differences observed in univariate baseline comparisons.

Three adiposity-related indicators exhibited divergent statistical significance across models: Waist-to-hip ratio showed a significant inverse association with T2DM in logistic regression (*P* < 0.05) but non-significant results in Cox regression (*P* = 0.99) and borderline protection in Fine-Gray models (*P* = 0.06).BMI ≥28.0 kg/m^2^ significantly elevated diabetes risk in logistic regression (*P* < 0.05), with attenuated non-significant effects in Cox regression (*P* = 0.40) and borderline significance in Fine-Gray models (*P* = 0.08).

Notably, hazard ratios for later enrollment years were markedly elevated in standard Cox regression, which arises from uneven follow-up time across recruitment cohorts. Such strong temporal association disappeared after adjustment for competing mortality events in Fine-Gray models.

**Fig. 1:**
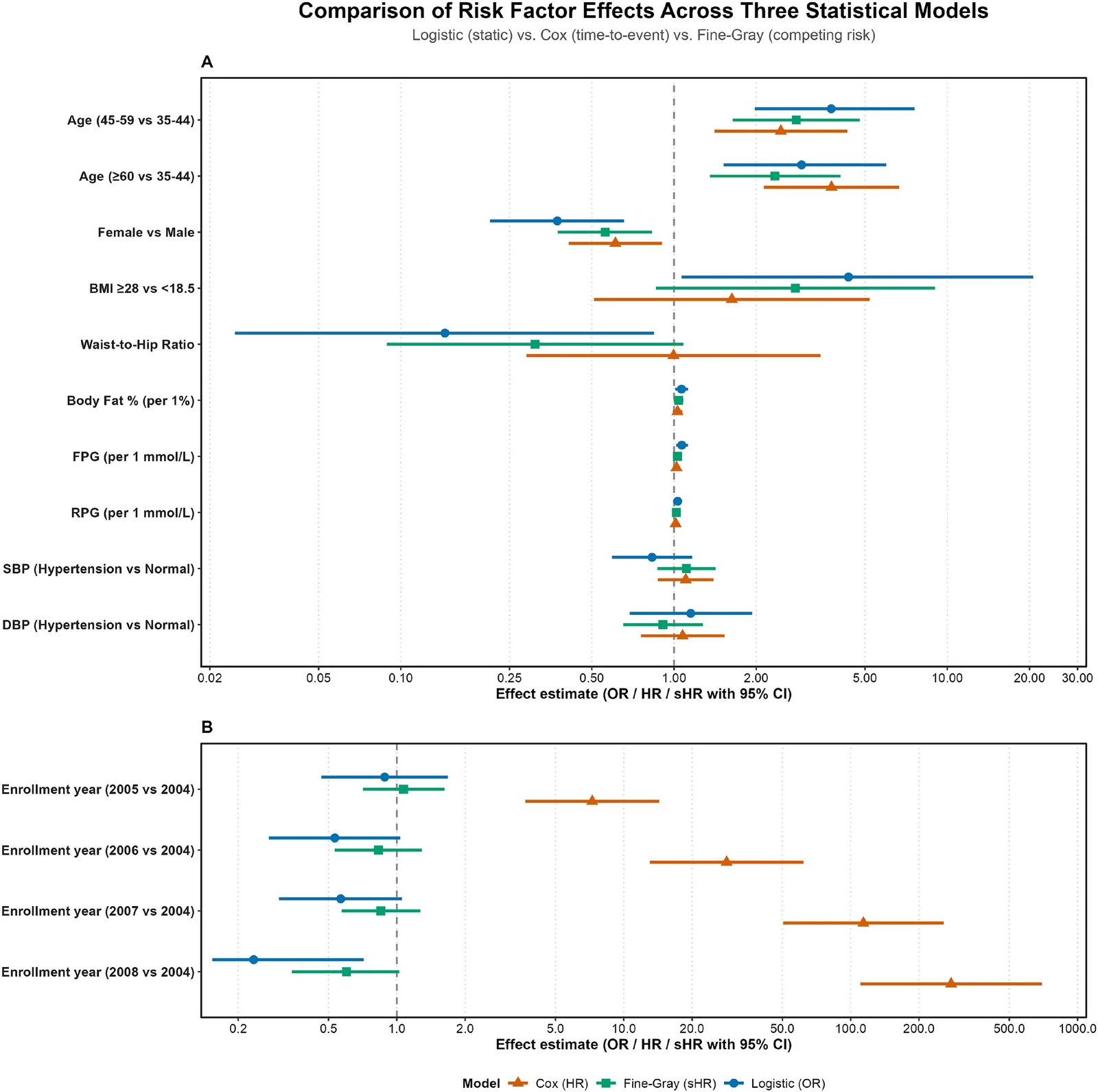
Fully adjusted effect estimates for predictors of incident type 2 diabetes among Chinese adults with impaired fasting glucose (5.6–6.9 mmol/L), Liuzhou CKB cohort (2004–2024). Panel A shows core risk factors, and Panel B shows enrollment year. Point estimates and 95% confidence intervals correspond to logistic regression (odds ratio; blue), Cox regression (hazard ratio; orange), and Fine-Gray competing-risk regression (subdistribution hazard ratio; green). Vertical dashed line = null effect (1). Abbreviations: BMI, body-mass index; FPG, fasting plasma glucose; RPG, random plasma glucose; SBP, systolic blood pressure; DBP, diastolic blood pressure; CKB, China Kadoorie Biobank. (For interpretation of the references to colour in this figure legend, the reader is referred to the web version of this article.)

Baseline demographic, anthropometric and glycemic indicators stratified by incident type 2 diabetes and mortality status are summarized in Table 1. Cross-model comparison of fully adjusted risk estimates across the three models is visualized in Fig. 1. Full numerical outputs for logistic, Cox and Fine-Gray regression models are provided in Supplementary Tables S1–S3.

**Table 1:** Baseline characteristics of Chinese adults with impaired fasting glucose recruited in Liuzhou, Guangxi (2004–2008), stratified by incident type 2 diabetes and all-cause mortality status (*N* = 913)).

Characteristics	All Participants (N = 913)	Progression of Diabetes		*P* value	Incidence of death		*P* value
		No (*n* = 526)	Yes (*n* = 387)		No (*n* = 668)	Yes (*n* = 245)	
Fasting Glucose (mmol/L)	6.14 ± 0.51	6.09 ± 5.70	6.21 ± 4.13	<0.01	6.13 ± 5.45	6.19 ± 4.08	0.02
Random Blood Glucose (mmol/L)	10.01 (8.80, 10.05)	9.30 (8.80, 10.2)	10.10 (9.10, 11.20)	<0.01	9.50 (8.90, 10.60)	9.70 (9.00, 10.90)	0.06
Body mass index (kg/m^2^)	24.84 ± 3.34	24.22 ± 3.35	25.67 ± 3.12	<0.01	24.70 (22.90, 26.80)	24.60 (22.30, 27.00)	0.60
Age (year)	57 (52, 65)	57 (51, 65)	57 (53, 64)	0.49	55 (50, 61)	66 (59, 71)	<0.01
Waist-to-Hip Ratio	0.92 ± 0.12	0.91 ± 0.12	0.93 ± 0.11	<0.01	0.92 ± 0.11	0.94 ± 0.92	0.06
Body Fat Percentage	29.53 ± 0.80	28.61 ± 7.84	30.79 ± 8.08	<0.01	30.11 ± 7.74	27.92 ± 8.48	<0.01
Systolic Blood Pressure (mmHg)	135.23 ± 21.63	132.99 ± 21.87	138.22 ± 20.84	<0.01	133.63 ± 21.46	139.67 ± 21.31	<0.01
Diastolic Blood P[ressire (mmHg)	75.80 ± 10.74	74.99 ± 11.17	76.89 ± 10.02	<0.01	76.18 ± 10.01	74.80 ± 12.45	0.01

Sex				0.08			
Male	347 (38.15%)	187 (35.55%)	160 (41.34%)		222 (33.28%)	125 (50.81%)	<0.01
Femal	566 (61.85%)	339 (64.45%)	227 (58.66%)		445 (66.72%)	121 (49.19%)	

Age Category (Year)							
Young (35–44)	70 (7.67%)	56 (10.65%)	14 (3.62%)	<0.01	66 (9.90%)	4 (1.63%)	<0.01
Middle (45–59)	448 (49.07%)	240 (45.63%)	208 (53.75%)		387 (58.02%)	61 (24.80%)	
Elderly (≥60)	395 (43.26%)	230 (43.73%)	165 (42.64%)		214 (32.08%)	181 (73.58%)	

Body mass indexI Category (kg/m^2^)							
Underweight (<18.5)	23 (2.52)%	19 (3.61%)	4 (1.03%)	<0.01	14 (2.10%)	9 (3.66%)	0.56
Normal (18.5–23.9)	344 (37.68%)	232 (44.11%)	112 (28.94%)		252 (37.78%)	92 (37.40%)	
Overweight (24–27.9)	390 (42.72%)	209 (39.73%)	181 (46.77%)		289 (43.33%)	101 (41.06)	
Obese (≥28)	156 (17.09%)	66 (12.55%)	90 (23.26%)		112 (16.79%)	44 (17.89%)	

Enrollment Year							
2004	62 (6.79%)	29 (5.51%)	33 (8.53%)	0.02	41 (6.15%)	21 (8.54%)	0.32
2005	205 (22.45%)	104 (19.77%)	101 (26.10%)		144 (21.59%)	61 (24.80%)	
2006	184 (20.15%)	112 (21.29%)	72 (18.60%)		134 (20.09%)	50 (20.33%)	
2007	377 (41.29%)	224 (42.59%)	153 (39.53%)		288 (43.18%)	89 (36.18%)	
2008	85 (9.31%)	57 (53 (10.84%)	28 (7.24%)		60 (9.00%)	25 (10.16%)	

Note: For continuous variables with non-normal distribution, data are presented as median with interquartile range (IQR).

## 4 Discussion

Our cross-model comparison revealed consistent risk-factor inferences for fasting glucose, body fat percentage, advanced age and female sex across logistic, Cox, and Fine-Gray competing-risk regression. These associations are broadly supported by previous prediabetes cohort studies (Richter et al., 2018; Ligthart et al., 2016; Mauricio et al., 2026).These biomarkers exert strong effects on incident type 2 diabetes, while their direct association with competing-event all-cause mortality was comparatively modest in this prediabetic cohort. Accordingly, differences in statistical handling of competing mortality exerted limited distortion on their risk estimates, yielding concordant findings across analytical approaches.

In contrast, divergent effect estimates across models were observed for BMI, waist-to-hip ratio, blood-pressure-related variables and enrollment-year variables. Such discrepancies cannot be attributed solely to high overall mortality within this long-term follow-up cohort. Instead, divergence arises when a given predictor influences both the primary outcome (type 2 diabetes) and the competing event (all-cause death). For these variables, logistic regression ignores time-to-event and competing death; standard Cox regression treats death as a censoring event and may overestimate risk; Fine-Gray competing-risk analysis formally accounts for death as a competing terminal event. When a predictor is strongly linked to mortality, assumptions underlying standard Cox and logistic models are more likely to be violated, leading to visible cross-model discrepancy. Predictors weakly associated with mortality remain robust across different regression frameworks, even under substantial cumulative mortality (Wolbers et al., 2014; Buzkova et al., 2019).

Elevated BMI showed a significant positive association only in logisitic regression;the association was attenuated and became non-significant in both Cox and Fine-Gray models. One tentative explanation is that individuals with obesity may face greater risk of premature mortality, which limits their opportunity to progress to type 2 diabetes. In contrast, waist-to-hip ratio exhibited a significant protective association only in static logistic regression. These divergent outputs reflect distinct pathogenic characteristics of different obesity phenotypes: total body fat accelerates glycemic deterioration, while central adiposity mainly increases premature mortality risk (Jayedi et al., 2022; Piché et al., 2020).

These observations underscore that prediabetes cohort studies with long-term follow-up should exercise caution when interpreting risk estimates from conventional Cox regression for exposures strongly predictive of mortality; competing-risk frameworks help to contextualize such divergent findings (Wolbers et al., 2014).Joint interpretation of multiple regression models is required to avoid biased risk inference in long cohorts with high competing mortality (Kunina et al., 2025).

### 4.1 Study limitations

Several limitations of this study should be acknowledged. First, all participants were recruited from a single regional Chinese cohort, which may restrict generalizability to other populations.Second, cross-model comparisons in the present study remain descriptive. Effect estimates derived from logistic, Cox, and Fine-Gray models have distinct statistical interpretations, and formal bootstrap-based testing for differences across these metrics is methodologically challenging; such formal comparisons could be considered in future investigations. Third, all-cause mortality was prespecified as the competing event for the Fine-Gray model in this analysis. Although this choice is methodologically appropriate, sensitivity analyses adopting disease-specific death endpoints may further explore the robustness of our findings.

## 5 Conclusions

Statistical model selection substantially influences risk factor inference for IFG cohorts with high competing mortality. Joint analysis of multiple complementary regression models is recommended to eliminate competing risk bias and generate reliable prediabetes risk conclusions.

## AI use

During manuscript preparation, the generative AI tool Doubao (ByteDance, https://www.doubao.com/) was used for English language polishing. All scientific content, statistical analyses, results and conclusions were reviewed, revised and verified by all authors. AI was not involved in study design, data collection, statistical analysis or result interpretation.

## CRediT authorship contribution statement

**Zhenzhen Lu:** Writing – original draft, Methodology, Formal analysis. **Chaoyun Li:** Data curation. **Qi Li:** Formal analysis, Data curation. **Jun Lv:** Supervision. **Canqing Yu:** Supervision, Methodology. **Dianjianyi Sun:** Writing – review & editing, Supervision. **Wei Mao:** Resources.

## Ethics declaration

Written informed consent to take part in the study and to publish the article has been obtained from all participants or their legal representatives. The privacy rights of participants have been observed.

This study was performed in compliance with relevant laws, regulatory frameworks and guidelines where the research took place.

This study was approved by the Ethics Review Committee of the Guangxi Zhuang Autonomous Region Center for Disease Control and Prevention (Approval No. GXCDCIRB 2022‑0044‑1).

## Funding

This work was supported by the Guangxi Medical and Health Appropriate Technology Development and Promotion Project (S2020010), grant (2023ZD0510100) from the Noncommunicable Chronic Diseases-National Science and Technology Major Project, grants (82192900, 82192901, 82192904, 81390540, 91846303) from the 10.13039/501100001809National Natural Science Foundation of China, grants (2016YFC0900500) from the 10.13039/501100012166National Key Research and Development Program of China, grants from the Kadoorie Charitable Foundation in Hong Kong and grants (202922/Z/16/Z, 088158/Z/09/Z, 104085/Z/14/Z) from 10.13039/100010269Wellcome Trust in the UK.

## Declaration of competing interest

The authors declare that they have no known competing financial interests or personal relationships that could have appeared to influence the work reported in this paper.

## Data Availability

The datasets are available from the corresponding author on reasonable request.
