# Supplementary material for: Risk factors for incident type 2 diabetes among adults with impaired fasting glucose: a comparison of three regression models accounting for competing mortality in Liuzhou, Guangxi, China
Source: Prev Med Rep. 2026 Sep 1;70:103616. doi: 10.1016/j.pmedr.2026.103616 (PMC13572089; doi:10.1016/j.pmedr.2026.103616)
Supplement: Supplementary file 1 [file mmc1.docx]

| Table S1.Associations of risk factors with incident type 2 diabetes from multivariate Logistic regression among Chinese adults with impaired fasting glucose in Liuzhou, Guangxi (2004–2008) | | | | | |
| --- | --- | --- | --- | --- | --- |
| Variable | β | SE | Wald χ² | OR | 95% CI for OR |
| Age (Ref: 35–44 year) |  |  |  |  |  |
| 45–59 | 1.32 | 0.33 | 15.62 | 3.76 | 2.00, 7.50 |
| ≥60 | 1.07 | 0.34 | 9.93 | 2.93 | 1.54, 5.90 |
| Gender (Female vs Male) | -0.98 | 0.28 | 12.25 | 0.37 | 0.21, 0.64 |
| Body mass index category (Ref:＜18.5 kg/m²) | — | — | — | — | — |
| 18.5–23.9 | 0.58 | 0.63 | 0.87 | 1.79 | 0.56, 7.01 |
| 24.0–27.9 | 1.01 | 0.67 | 2.30 | 2.76 | 0.79, 11.51 |
| ≥28.0 | 1.47 | 0.74 | 3.95 | 4.35 | 1.08, 20.39 |
| Systolic blood pressure (Ref: Normal<140mmHg) |  |  |  |  |  |
| Hypertension(1) | -0.18 | 0.166 | 1.26 | 0.83 | 0.60, 1.15 |
| Diastolic blood pressure (Ref: Normal<90mmHg) |  |  |  |  |  |
| Hypertension(1) | 0.14 | 0.26 | 0.30 | 1.15 | 0.70, 1.91 |
| Body Fat Percentage(per 1% increment) | 0.06 | 0.02 | 9.11 | 1.06 | 1.02, 1.11 |
| Fasting plasma glucose(per 1 mmol/L increase) | 0.06 | 0.019 | 12.49 | 1.07 | 1.03, 1.11 |
| Random plasma glucose(per 1 mmol/L increase) | 0.03 | 0.01 | 34.57 | 1.03 | 1.02, 1.04 |
| Waist-to-Hip Ratio | -1.93 | 0.89 | 4.64 | 0.14 | 0.02, 0.83 |
| Enrollment Year (Ref: 2004 ) | — | — | — | — | — |
| 2005 | -0.12 | 0.32 | 0.15 | 0.88 | 0.47, 1.65 |
| 2006 | -0.63 | 0.33 | 3.61 | 0.53 | 0.28, 1.02 |
| 2007 | -0.57 | 0.31 | 3.39 | 0.57 | 0.31, 1.04 |
| 2008 | -1.09 | 0.38 | 8.19 | 0.33 | 0.16, 0.70 |
| Note:β, regression coefficient; SE, standard error; Wald χ², Wald chi‑square statistic; OR, odds ratio; 95% CI, 95% confidence interval; Ref., reference group. | | | | | |
