# Supplementary material for: Risk factors for incident type 2 diabetes among adults with impaired fasting glucose: a comparison of three regression models accounting for competing mortality in Liuzhou, Guangxi, China
Source: Prev Med Rep. 2026 Sep 1;70:103616. doi: 10.1016/j.pmedr.2026.103616 (PMC13572089; doi:10.1016/j.pmedr.2026.103616)
Supplement: Supplementary file 2 [file mmc2.docx]

| Table S2.Multivariate Cox proportional hazards regression for predictors of incident type 2 diabetes among Chinese adults with impaired fasting glucose in Liuzhou, Guangxi (2004–2008) | | | | | |
| --- | --- | --- | --- | --- | --- |
| Variable | β | SE | Wald χ² | HR | 95% CI for HR |
| Age (Ref: 35–44 year) |  |  |  |  |  |
| 45–59 | 0.90 | 0.28 | 7.09 | 2.46 | 1.42, 4.25 |
| ≥60 | 1.33 | 0.28 | 21.72 | 3.77 | 2.16, 6.59 |
| Gender (Female vs Male) | -0.49 | 0.19 | 6.51 | 0.61 | 0.42, 0.89 |
| Body mass index(Ref:＜18.5 kg/m²) | — | — | — | — | — |
| 18.5–23.9 | 0.26 | 0.53 | 0.24 | 1.29 | 0.46, 3.64 |
| 24.0–27.9 | 0.41 | 0.55 | 0.57 | 1.51 | 0.52, 4.43 |
| ≥28.0 | 0.48 | 0.59 | 0.69 | 1.63 | 0.52, 5.13 |
| Systolic blood pressure (Ref: Normal<140mmHg) |  |  |  |  |  |
| Hypertension(1) | 0.10 | 0.11 | 0.76 | 1.10 | 0.88, 1.38 |
| Diastolic blood pressure(Ref: Normal<90mmHg) |  |  |  |  |  |
| Hypertension(1) | 0.07 | 0.17 | 0.18 | 1.08 | 0.77, 1.51 |
| Body Fat Percentage(per 1% increment) | 0.03 | 0.01 | 4.68 | 1.03 | 1.00, 1.06 |
| Fasting plasma glucose(per 1 mmol/L increase) | 0.02 | 0.01 | 13.65 | 1.02 | 1.01, 1.03 |
| Random plasma glucose(per 1 mmol/L increase) | 0.01 | 0.00 | 28.62 | 1.01 | 1.01, 1.02 |
| Waist-to-Hip Ratio | -0.01 | 0.63 | 0.00 | 1.00 | 0.29, 3.40 |
| Enrollment Year (Ref:2004) | — | — | — | — | — |
| 2005 | 1.98 | 0.34 | 34.27 | 7.26 | 3.74, 14.11 |
| 2006 | 3.35 | 0.39 | 73.57 | 28.41 | 13.22, 61.04 |
| 2007 | 4.73 | 0.41 | 135.02 | 113.78 | 51.20, 252.85 |
| 2008 | 5.62 | 0.46 | 147.95 | 277.10 | 111.96, 685.83 |
| Note:β, regression coefficient; SE, standard error; Wald χ², Wald chi-square statistic; HR, hazard ratio; 95% CI, 95% confidence interval; Ref., reference group; mmHg, millimeters of mercury; mmol/L, millimoles per liter. | | | | | |
