# Supplementary material for: Risk factors for incident type 2 diabetes among adults with impaired fasting glucose: a comparison of three regression models accounting for competing mortality in Liuzhou, Guangxi, China
Source: Prev Med Rep. 2026 Sep 1;70:103616. doi: 10.1016/j.pmedr.2026.103616 (PMC13572089; doi:10.1016/j.pmedr.2026.103616)
Supplement: Supplementary file 3 [file mmc3.docx]

| Table S3. Associations of risk factors with incident type 2 diabetes from univariate and multivariable Fine–Gray competing risk regression models among Chinese adults with impaired fasting glucose in Liuzhou, Guangxi (2004–2008) | | | |
| --- | --- | --- | --- |
| Variable | Model 1: Univariate HR (95% CI) | Model 2: Partially Adjusted sHR (95% CI) | Model 3: Fully Adjusted sHR (95% CI) |
| Age (Ref: 35–44 year) |  |  |  |
| 45–59 | 2.86 (1.68, 4.87) | 2.83 (1.67, 4.81) | 2.80 (1.66, 4.72) |
| ≥60 | 2.47 (1.44, 4.21) | 2.39 (1.39, 4.11) | 2.34 (1.37, 4.02) |
| Gender (Female vs Male) | 0.84 (0.69, 1.03) | 0.56 (0.39, 0.79) | 0.56 (0.38, 0.82) |
| Body mass indexI (Ref: <18.5 kg/m²) |  |  |  |
| 18.5–23.9 | — | 1.53 (0.57, 4.11) | 1.65 (0.59, 4.62) |
| 24–27.9 | — | 1.96 (0.70, 5.55) | 2.35 (0.80, 6.94) |
| ≥28 | — | 2.19 (0.72, 6.62) | 2.78 (0.87, 8.91) |
| Systolic blood pressure (Ref: Normal<140mmHg) |  |  |  |
| Hypertension | — | 1.16 (0.93, 1.46) | 1.11 (0.88, 1.40) |
| Diastolic blood pressure(Ref: Normal<90mmHg) |  |  |  |
| Hypertension | — | 0.89 (0.64, 1.24) | 0.91 (0.66, 1.26) |
| Body Fat Percentage (per 1% increment) | — | 1.04 (1.01, 1.06) | 1.04 (1.01, 1.07) |
| Fasting plasma glucose (per 1 mmol/L increase) | — | — | 1.03 (1.01, 1.04) |
| Random plasma glucose (per 1 mmol/L increase) | — | — | 1.02 (1.01, 1.02) |
| Waist-to-Hip Ratio | — | — | 0.31 (0.09, 1.07) |
| Enrollment Year (Ref: 2004) |  |  |  |
| 2005 | — | — | 1.07 (0.72, 1.60) |
| 2006 | — | — | 0.83 (0.54, 1.27) |
| 2007 | — | — | 0.85 (0.58, 1.25) |
| 2008 | — | — | 0.60 (0.35, 1.01) |
| Model 1: Univariate analysis. Model 2 (Partially Adjusted): Adjusted for age, sex and Body mass indexI category. Model 3 (Fully Adjusted): Adjusted for all variables listed in the table. sHR, subdistribution hazard ratio; CI, confidence interval;  A confidence interval crossing 1.0 indicates a non-statistically significant association at the α=0.05 level. A dash (—) indicates that category-specific estimates were not calculated in the univariable model.  Ref., reference group. | | | |
